# Lipid exchange among electroneutral Sulfo-DIBMA nanodiscs is independent of ion concentration

**DOI:** 10.1515/hsz-2022-0319

**Published:** 2023-03-17

**Authors:** Loretta Eggenreich, Carolyn Vargas, Cenek Kolar, Sandro Keller

**Affiliations:** Biophysics, Institute of Molecular Biosciences (IMB), NAWI Graz, University of Graz, Humboldtstr. 50/III, A-8010 Graz, Austria; Field of Excellence BioHealth, University of Graz, Graz, Austria; BioTechMed-Graz, Graz, Austria; Glycon Biochemicals GmbH, Im Biotechnologiepark TGZ 1, D-14943 Luckenwalde, Germany

**Keywords:** kinetics, polymer-encapsulated nanodiscs, stopped flow, time-resolved FRET

## Abstract

Polymer-encapsulated nanodiscs enable membrane proteins to be investigated within a native-like lipid-bilayer environment. Unlike other bilayer-based membrane mimetics, these nanodiscs are equilibrium structures that permit lipid exchange on experimentally relevant timescales. Therefore, examining the kinetics and mechanisms of lipid exchange is of great interest. Since the high charge densities of existing anionic polymers can interfere with protein–protein and protein–lipid interactions as well as charge-sensitive analysis techniques, electroneutral nanodisc-forming polymers have been recently introduced. However, it has remained unclear how the electroneutrality of these polymers affects the lipid-exchange behavior of the nanodiscs. Here, we use time-resolved Förster resonance energy transfer to study the kinetics and the mechanisms of lipid exchange among nanodiscs formed by the electroneutral polymer Sulfo-DIBMA. We also examine the role of coulombic repulsion and specific counterion association in lipid exchange. Our results show that Sulfo-DIBMA nanodiscs exchange lipids on a similar timescale as DIBMA nanodiscs. In contrast with nanodiscs made from polyanionic DIBMA, however, the presence of mono- and divalent cations does not influence lipid exchange among Sulfo-DIBMA nanodiscs, as expected from their electroneutrality. The robustness of Sulfo-DIBMA nanodiscs against varying ion concentrations opens new possibilities for investigating charge-sensitive processes involving membrane proteins.

## 1 Introduction

Polymer-encapsulated nanodiscs are self-assembling discoidal nanoparticles in which a belt of amphiphilic copolymer chains encapsulates a central lipid-bilayer core that can harbor a membrane protein or membrane-protein complex (Dörr et al. 2016; Knowles et al. 2009). These nanodiscs thus allow membrane proteins to be investigated within a native-like lipid-bilayer environment, thereby enabling the study of protein functions that depend on protein–lipid interactions (Danielczak and Keller 2020).

The formation and physicochemical properties of polymer-encapsulated nanodiscs are strongly influenced by polymer properties such as chemical composition, chain length, and—most notably—charge (Glueck et al. 2022; Hoffmann et al. 2021a; Scheidelaar et al. 2016). For example, the well-characterized polymers DIBMA (diisobutylene/maleic acid copolymer) and SMA (styrene/maleic acid copolymer) carry negative charges due to the carboxylate groups in their maleic acid units (Danielczak and Keller 2018; Glueck et al. 2022). Consequently, in nanodiscs formed from DIBMA or SMA, the high charge densities of these polymers can lead to unspecific polymer–protein and polymer–lipid interactions, which may interfere with the protein–protein and protein–lipid interactions of interest (Glueck et al. 2022). Therefore, a number of charge-sensitive analytical and preparative techniques cannot be used with DIBMA-and SMA-based nanodiscs (Glueck et al. 2022). To overcome these major issues, electroneutral polymers have recently been introduced. For instance, the electroneutral zSMA polymers designed by Fiori et al. (2017) carry zwitterionic phosphocholine groups. zSMA polymers are able to solubilize lipids and extract membrane proteins into lipid-bilayer nanodiscs, but their multistep synthesis is rather complicated (Glueck et al. 2022). To address this issue, our group has recently introduced the two electroneutral polymers Sulfo-DIBMA and Sulfo-SMA, which can be synthesized from common polymer backbones in a simple two-step procedure (Glueck et al. 2022).

These electroneutral Sulfo-polymers can be prepared simply by attaching zwitterionic sulfobetaine groups to the commercially available polymers DIBMA and SMA to abolish the negative charges on the two parent polymers (Glueck et al. 2022). A similar SMA-derivative has already been used in previous studies (Hoffmann et al. 2021b). Both electroneutral Sulfo-polymers, but especially Sulfo-DIBMA, outperform their polyanionic analogs DIBMA and SMA (Glueck et al. 2022). For instance, formation of Sulfo-DIBMA nanodiscs proceeds smoothly at various ion concentrations, even at 80 mM Mg^2+^ and Ca^2+^, and the nanodiscs remain colloidally stable at these ion concentrations. Moreover, Sulfo-DIBMA does not interfere with charge-sensitive interactions between proteins and lipids and, therefore, enables such interactions to be detected by microfluidic diffusional sizing (Glueck et al. 2022). Also, Sulfo-DIBMA is compatible with cell-free membrane-protein translation and can extract membrane proteins of different folds, sizes, and oligomeric states from human cells, even while preserving delicate noncovalent protein–protein interactions (Glueck et al. 2022). Furthermore, the acyl-chain packing of encapsulated lipids is perturbed even less by Sulfo-DIBMA than by DIBMA (Glueck et al. 2022). Finally, Sulfo-DIBMA nanodiscs have successfully been used in cryogenic electron microscopy (cryoEM) studies of endogenous eukaryotic membrane proteins, thereby outperforming other nanodisc-forming polymers in terms of protein-extraction efficiency and sample quality (Janson et al. 2022).

An inherent property of polymer-encapsulated nanodiscs is that they are equilibrium structures and can exchange lipids, as well as proteins, among themselves and with other lipid-bilayer systems (Broecker et al. 2017; Cuevas Arenas et al. 2016; Dörr et al. 2014; Hazell et al. 2016). As control over the lipid composition in membrane mimetics is important for their application in membrane-protein studies, examining the kinetics and mechanisms of lipid exchange is of great interest (Cuevas Arenas et al. 2017). Lipid exchange among polymer-encapsulated nanodiscs, represented by the lipid-concentration-dependent rate coefficient *k*_obs_, occurs through two main mechanisms (Cuevas Arenas et al. 2017; Danielczak and Keller 2020). The first mechanism is diffusional exchange, which includes desorption and inter-particle diffusion of lipid monomers through the aqueous phase surrounding the nanodiscs. This mechanism is represented by the rate coefficient *k*_diff_ (Nichols 1985; Nichols and Pagano 1981, 1982). The second mechanism is collisional lipid exchange, including binary and ternary collisions, represented by the rate coefficients *k*_bi_ and *k*_ter_. These collisions lead to transient fusion of nanodiscs, allowing *en masse* lipid exchange (Danielczak and Keller 2018; Fullington et al. 1990; Fullington and Nichols 1993; Grethen et al. 2018; Jones and Thompson 1990; Nichols 1988).

Lipid exchange among nanodiscs encapsulated by polyanionic polymers has been reported to be accelerated by the presence of mono-and divalent ions (Danielczak and Keller 2018; Grethen et al. 2018). This can be rationalized by the fact that cations can shield the negative charges on the polymer belt of each nanodisc, reducing the electrostatic repulsions between nanodiscs (Danielczak and Keller 2018, 2020; Grethen et al. 2018). With these observations and rationale in mind, we hypothesized that, for nanodiscs encapsulated by the electroneutral polymer Sulfo-DIBMA, lipid exchange should be unaffected by changes in ion concentration. To test this hypothesis, we investigated lipid exchange among Sulfo-DIBMA nanodiscs using time-resolved Förster resonance energy transfer (TR-FRET) spectroscopy.

## 2 Results

### 2.1 The dominant lipid-exchange mechanism depends on lipid concentration

To investigate the lipid-exchange mechanisms of electroneutral Sulfo-DIBMA nanodiscs, we performed TR-FRET measurements with fluorescently labeled Sulfo-DIBMA nanodiscs using a stopped-flow apparatus. Briefly, this method relies on monitoring changes in the FRET efficiency between fluorescently labeled donor and acceptor lipids (Danielczak and Keller 2020). To this end, a stopped-flow apparatus is used to mix nanodiscs containing low mole fractions (typically, ∼2 mol%) of both donor-labeled lipids and acceptor-labeled lipids with an excess of unlabeled lipid-bilayer nanodiscs. Once mixed, the two nanodisc populations exchange lipids with each other, so that the two fluorescently labeled lipid species become diluted across all nanodiscs. As a consequence, the average distance between donor and acceptor lipids increases, which leads to an increase in the fluorescence emission intensity of the donor fluorophore, that is, donor dequenching. A series of such dequenching traces measured at different concentrations of unlabeled nanodiscs can then be analyzed by non-linear least-squares fitting (Kemmer and Keller 2010) to determine the rate coefficients characterizing various lipid-exchange mechanisms (Danielczak and Keller 2020; Kemmer and Keller 2010).

**Figure 1:**
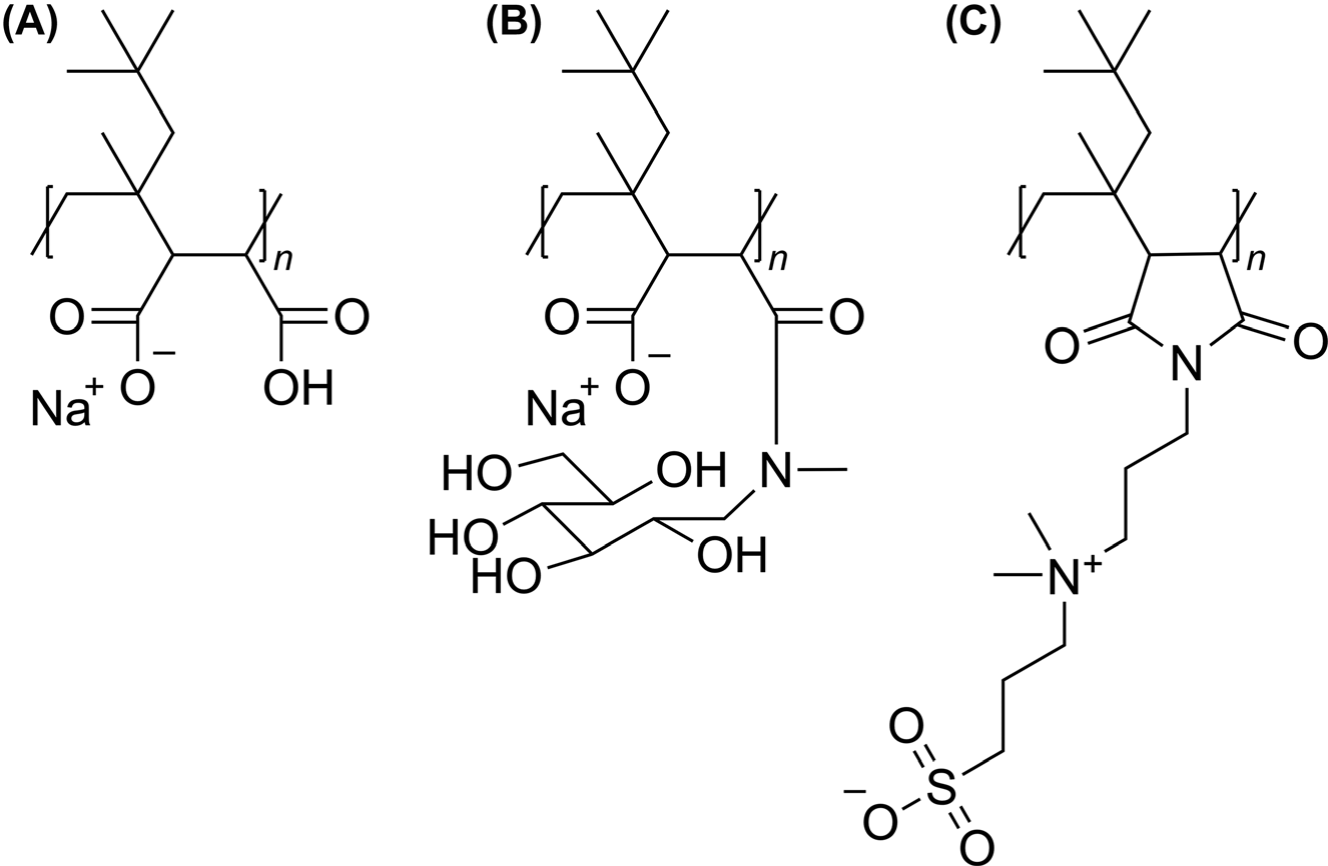
Chemical structures of (A) DIBMA (Oluwole et al. 2017), (B) Glyco-DIBMA (Danielczak et al. 2022), and (C) Sulfo-DIBMA (Glueck et al. 2022). DIBMA and Glyco-DIBMA carry anionic maleic acid and meglumine maleamidic acid units, respectively. By contrast, Sulfo-DIBMA has electroneutral, zwitterionic sulfobetaine maleimide units.

**Figure 2:**
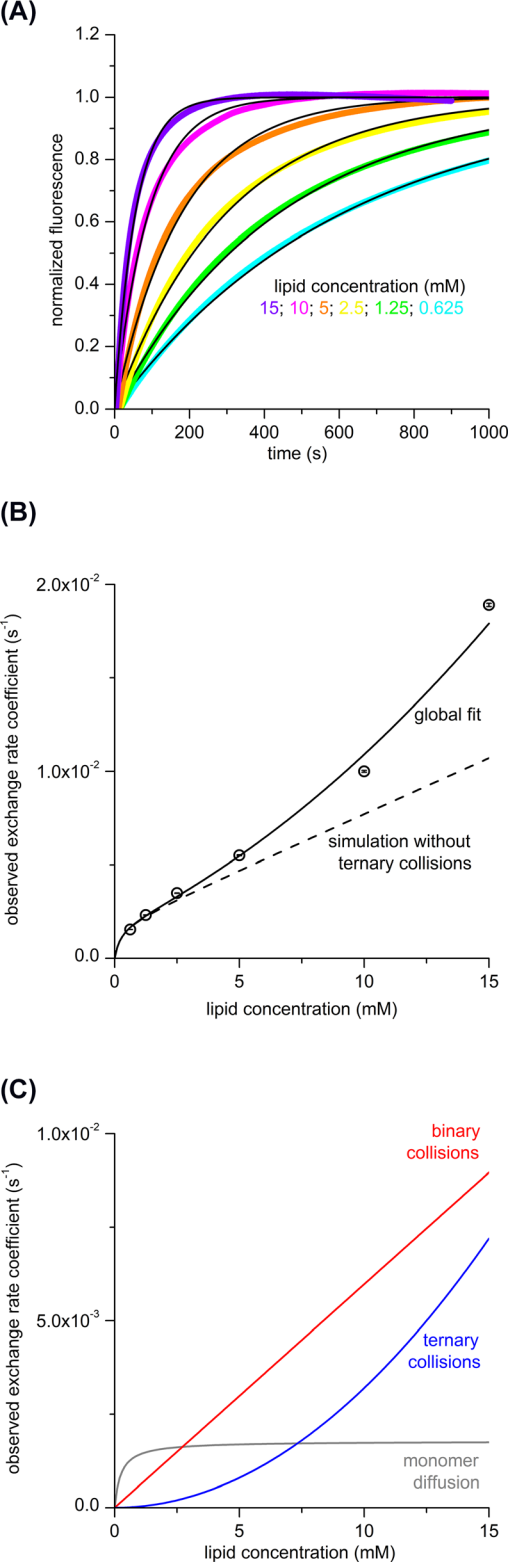
Lipid exchange among Sulfo-DIBMA nanodiscs occurs through both monomer diffusion and nanodisc collisions. (A) TR-FRET data showing normalized fluorescence intensity at a wavelength of (528 ± 15) nm versus time after mixing labeled nanodiscs at a final lipid concentration of *c*_L_° = 0.25 mM with unlabeled nanodiscs at a final lipid concentration of 0.625 mM ≤ *c*_L_ ≤ 15.0 mM. Shown are experimental data (colored lines, averaged over five measurement runs with 10,000 data points per run) and a global fit (black lines) according to Equations (2) and (3). (B) Overall rate coefficients of lipid exchange derived from TR-FRET. Shown are results from local fits (circles), a global fit (solid line), and a prediction based on the global fit but neglecting ternary collisions (dashed line) according to Equations (2) and (3). Error bars (within circles) indicate 95% confidence intervals for local fits. (C) Contributions of diffusional and collisional lipid exchange to the overall rate coefficient as functions of lipid concentration according to Equation (2).

To facilitate comparison with other nanodisc-forming polymers (Figure 1), we chose the same lipids as in previous studies with nanodiscs made from DIBMA (Danielczak and Keller 2018) or the more hydrophobic DIBMA derivative Glyco-DIBMA (Danielczak et al. 2022). Specifically, we used 1,2-dimyristoyl-*sn*-glycero-3-phosphocholine (DMPC) as unlabeled lipid and 1,2-dihexadecanoyl-*sn*-glycero-3-phosphoethanolamine (NBD-PE) and *N*-(lissamine rhodamine B-sulfonyl)-1,2-dihexadecanoyl-*sn*-glycero-3-phosphoethanolamine (Rh-PE) as fluorescently labeled lipids carrying FRET donors and acceptors, respectively. DMPC is a zwitterionic, fully saturated phospholipid. Given that lipid exchange takes place predominantly through nanodisc collisions rather than monomer diffusion at typical experimental lipid concentrations (see below), we expect the major conclusions of the present study to carry over to other lipid species. Moreover, we chose lipid concentrations similar to those in the above-mentioned studies (Danielczak and Keller 2018, Danielczak et al. 2022). Thus, the final concentrations of unlabeled lipids after mixing in the stopped-flow apparatus ranged from 0.625 mM to 15.0 mM (Figure 2A).

Our results reveal that diffusional lipid exchange dominates at lower lipid concentrations, whereas collisional lipid exchange dominates at higher concentrations (Figure 2B, C). The concentration-dependent overall rate coefficient *k*_obs_ and the concentration-independent rate coefficients *k*_diff_, *k*_bi_, and *k*_ter_ were determined by nonlinear least-squares fitting as previously described (Kemmer and Keller 2010). Thus, we could extract the contributions of each lipid-exchange mechanism to the overall lipid exchange (Danielczak and Keller 2020; Kemmer and Keller 2010) (Figure 2B, C). At lipid concentrations below 2.5 mM, diffusional exchange (represented by *k*_diff_) was the dominant lipid-exchange process, whereas binary collisions (*k*_bi_) made the largest contribution above 2.5 mM, with ternary collisions (*k*_ter_) also noticeably contributing at lipid concentrations above 2.5 mM (Figure 2B, C).

**Figure 3:**
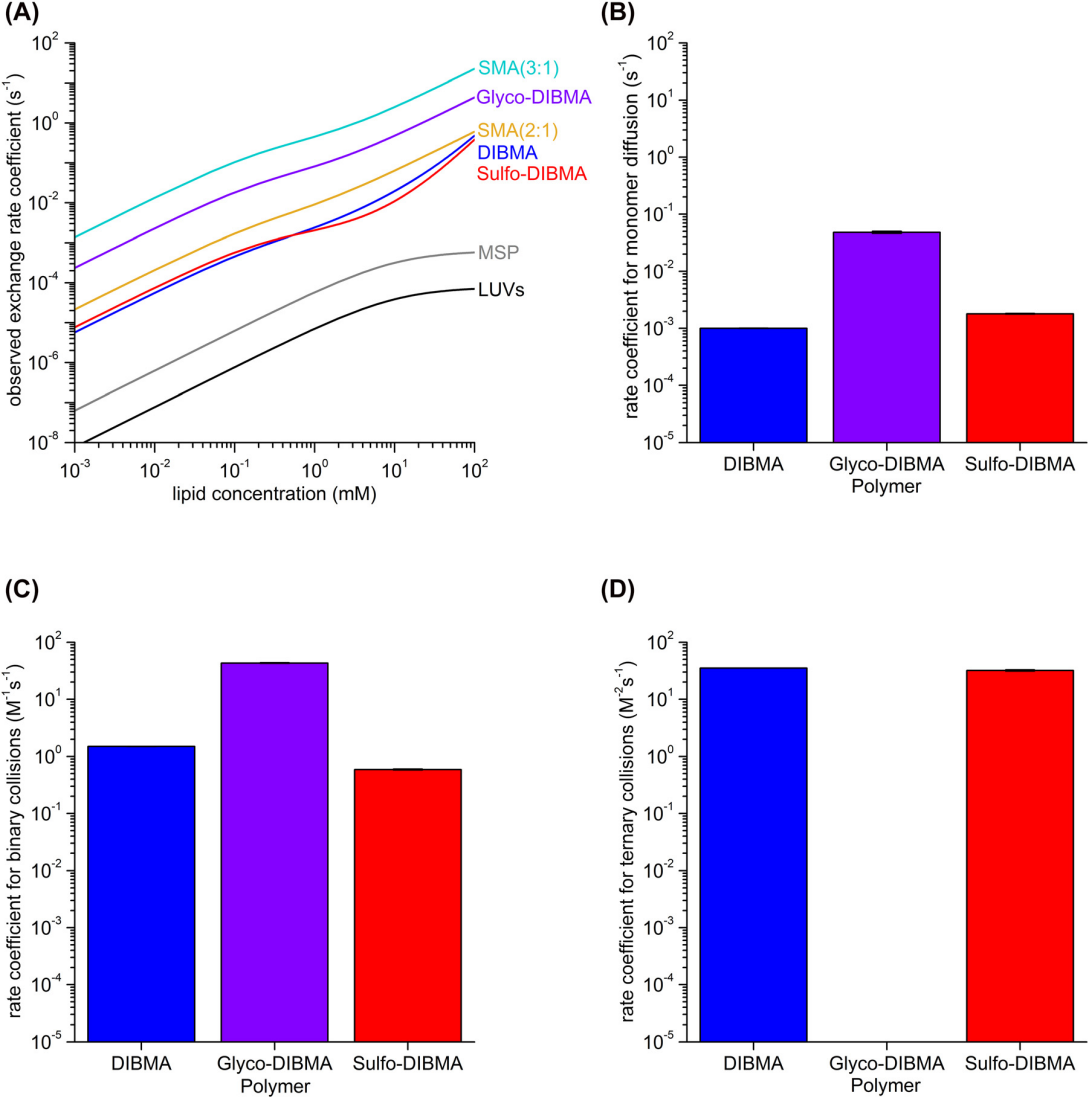
Lipid exchange among Sulfo-DIBMA nanodiscs is slower than in most other polymer-encapsulated nanodiscs but faster than in LUVs and MSP nanodiscs. (A) Overall lipid-exchange rate coefficients as functions of lipid concentration for nanodiscs encapsulated by DIBMA (Danielczak and Keller 2018), Glyco-DIBMA (Danielczak et al. 2022), Sulfo-DIBMA (this work), SMA (2:1) (Grethen et al. 2018), SMA (3:1) (Cuevas Arenas et al. 2017), or membrane-scaffold protein (Nakano et al. 2009) and for large unilamellar vesicles (LUVs) (Nakano et al. 2007). Polymer-encapsulated nanodiscs were studied at 30 °C and an ionic strength of 200 mM, while membrane-scaffold-protein nanodiscs and large unilamellar vesicles were studied at 27 °C and an ionic strength of 150 mM. In all cases, the major lipid component was DMPC. (B–D) Detailed comparison of (B) diffusional, (C) binary collisional, and (D) ternary collisional lipid-exchange rate coefficients of DIBMA (Danielczak and Keller 2018), Glyco-DIBMA (Danielczak et al. 2022), and Sulfo-DIBMA (this work) nanodiscs. Error bars indicate 95% confidence intervals.

The overall lipid-exchange kinetics of Sulfo-DIBMA nanodiscs (given by *k*_obs_) is on the same order of magnitude as that of DIBMA nanodiscs across a broad range of lipid concentrations (Figure 3A, Table 1). This similarity applies to all three mechanisms of lipid exchange, as follows. First, the *k*_diff_ value for Sulfo-DIBMA nanodiscs (*k*_diff_ = (1.8 × 10^−3^ ± 2 × 10^−5^) s^−1^) is close to that determined for DIBMA nanodiscs (*k*_diff_ = (1.0 × 10^−3^ ± 0.5 × 10^−5^) s^−1^) (Danielczak and Keller 2018) (Figure 3B, Table 1). Second, regarding collisional lipid exchange, the *k*_bi_ value found for Sulfo-DIBMA nanodiscs (*k*_bi_ = (0.59 ± 0.01) M^−1^ s^−1^) is less than a factor of three lower than that for DIBMA nanodiscs (*k*_bi_ = (1.5 ± 0.004) M^−1^ s^−1^) under similar conditions (Danielczak and Keller 2018) (Figure 3C, Table 1). Third, the *k*_ter_ value for Sulfo-DIBMA nanodiscs (*k*_ter_ = (32.0 ± 1.1) M^−2^ s^−1^) is also similar to that for DIBMA nanodiscs (*k*_ter_ = (35.1 ± 0.3) M^−2^ s^−1^) (Danielczak and Keller 2018) (Figure 3D, Table 1).

There are substantial differences, however, between Sulfo-DIBMA and Glyco-DIBMA in the kinetics of individual lipid-exchange mechanisms. On the one hand, diffusional lipid exchange among Sulfo-DIBMA nanodiscs (*k*_diff_ = (1.8 × 10^−3^ ± 2 × 10^−5^) s^−1^) and exchange through binary collisions (*k*_bi_ = (0.59 ± 0.01) M^−1^ s^−1^) were both more than an order of magnitude slower than the respective processes for Glyco-DIBMA nanodiscs (*k*_diff_ = (4.8 × 10^−2^ ± 2 × 10^−3^) s^−1^, *k*_bi_ = (43.1 ± 0.5) M^−1^ s^−1^) (Danielczak et al. 2022) (Figure 3B, C, Table 1). On the other hand, ternary collisions contributed noticeably to the collisional lipid exchange among Sulfo-DIBMA nanodiscs at higher lipid concentrations, whereas they were not observed for Glyco-DIBMA nanodiscs (Danielczak et al. 2022) (Figure 3D, Table 1).

In summary, we found the lipid exchange behavior of Sulfo-DIBMA nanodiscs to be similar to that of DIBMA nanodiscs across a broad range of lipid concentrations but different from that of Glyco-DIBMA nanodiscs (Figure 3A, Table 1) (Danielczak et al. 2022). For an overall comparison, we include in Figure 3A also the lipid-exchange kinetics previously determined for more hydrophobic SMA nanodiscs (Cuevas Arenas et al. 2017; Grethen et al. 2018), for nanodiscs encapsulated by a membrane-scaffold protein (MSP) (Nakano et al. 2009), and for large unilamellar vesicles (Nakano et al. 2007).

**Table 1:** Best-fit values of diffusional rate coefficients, binary collisional rate coefficients, and ternary collisional rate coefficients with their corresponding 95% confidence intervals for DMPC nanodiscs encapsulated by DIBMA (Danielczak and Keller 2018), Glyco-DIBMA (Danielczak et al. 2022), and Sulfo-DIBMA (this work) at various NaCl concentrations.

Polymer	c(NaCl) (mM)	*k*_diff_ (s^−1^)	*k*_bi_ (M^−1^ s^−1^)	*k*_ter_ (M^−2^ s^−1^)
DIBMA (Danielczak and Keller 2018)	100	2.2 × 10^−4^ ± 1 × 10^−6^	0.12 ± 0.001	13.9 ± 0.1
	200	1.0 × 10^−3^ ± 0.5 × 10^−5^	1.5 ± 0.004	35.1 ± 0.3
	600	1.7 × 10^−2^ ± 1 × 10^−4^	33.5 ± 0.1	433 ± 6
Glyco-DIBMA (Danielczak et al. 2022)	200	4.8 × 10^−2^ ± 2 × 10^−3^	43.1 ± 0.5	—
Sulfo-DIBMA (this work)	200	1.8 × 10^−3^ ± 2 × 10^−5^	0.59 ± 0.01	32.0 ± 1.1

### 2.2 Increasing ionic strength does not affect lipid exchange among Sulfo-DIBMA nanodiscs

As stated in the Introduction, we hypothesized that, for nanodiscs encapsulated by the electroneutral polymer Sulfo-DIBMA, lipid exchange should be unaffected by changes in ionic strength and, thus, salt concentration. To test this hypothesis, we first measured lipid exchange among Sulfo-DIBMA nanodiscs at three different NaCl concentrations. As in the previous experiments, lipid exchange was analyzed by measuring TR-FRET with a stopped-flow apparatus. To this end, Sulfo-DIBMA nanodiscs were prepared in aqueous buffers containing 50, 200, or 500 mM NaCl.

**Figure 4:**
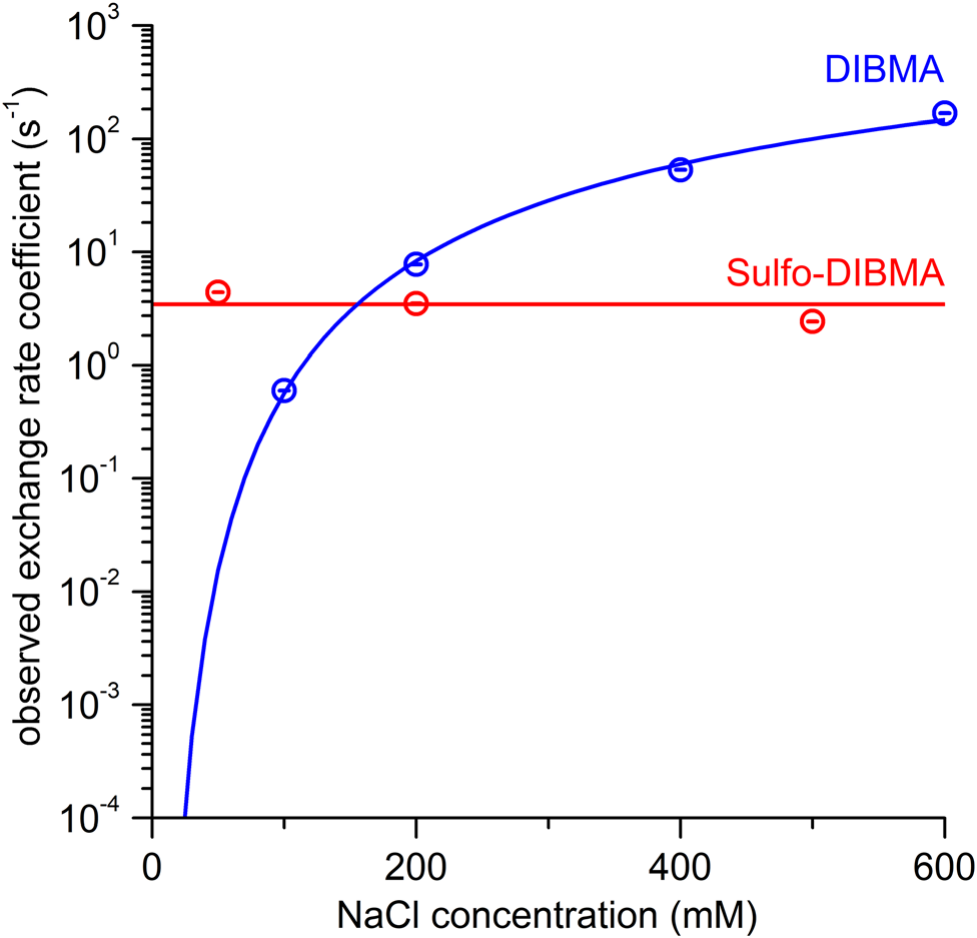
Ionic strength does not affect lipid exchange among Sulfo-DIBMA nanodiscs. Observed rate coefficients characterizing overall lipid exchange (*k*_obs_) as functions of NaCl concentration for Sulfo-DIBMA nanodiscs (this work) and DIBMA nanodiscs (Danielczak and Keller 2018). Circles show experimental data, while solid lines represent fits according to Equation (4). Error bars (within circles) indicate 95% confidence intervals.

Indeed, lipid exchange among electroneutral Sulfo-DIBMA nanodiscs was not noticeably affected by changes in ionic strength. Specifically, the overall observed rate coefficient for lipid exchange (*k*_obs_) varied by less than a factor of two across all NaCl concentrations tested (Figure 4). These results contrast strongly with those previously reported for DIBMA nanodiscs (Figure 4), which show a >10-fold increase in *k*_obs_ when the NaCl concentration is increased from 100 to 200 mM (Danielczak and Keller 2018). In comparison, the variation in *k*_bi_ observed for Sulfo-DIBMA nanodiscs at different ionic strengths is insignificant, which supports our hypothesis that lipid exchange should be unaffected by changes in ionic concentration for electroneutral Sulfo-DIBMA nanodiscs.

### 2.3 Ca^2+^ and Mg^2+^ do not affect lipid exchange among Sulfo-DIBMA nanodiscs

Having shown that the lipid exchange among Sulfo-DIBMA nanodiscs is not affected by monovalent ions, we aimed to see if this could be extended to the divalent cations Mg^2+^ and Ca^2+^, as they are particularly physiologically relevant. These two cations have been found to reduce coulombic repulsion among polymer-encapsulated nanodiscs much more efficiently than monovalent ions (Bretti et al. 2005; Danielczak et al. 2019; Kläning and Østerby 1976).This strong effect is due to counterion association and, therefore, goes way beyond a simple increase in coulombic screening resulting from the higher charge density of divalent as compared with monovalent metal ions (Bergethon 1998; Danielczak et al. 2019; Emyr Alun Moelwyn-Hughes 1963). In the case of polyanionic polymers such as DIBMA, Mg^2+^ and Ca^2+^ associate avidly and specifically with the polymer’s carboxylate groups, thereby reducing the effective charge on the nanodiscs and accelerating collisional lipid exchange (Bergethon 1998; Danielczak et al. 2019; Emyr Alun Moelwyn-Hughes 1963). Therefore, we were keen to see whether the lipid-exchange behavior of the electroneutral Sulfo-DIBMA nanodiscs would be insensitive even to divalent ions. To this end, we measured the lipid exchange among Sulfo-DIBMA nanodiscs in the presence of 5 mM Mg^2+^ or Ca^2+^, in order to be comparable with previous results obtained for DIBMA nanodiscs (Danielczak et al. 2019).

**Figure 5:**
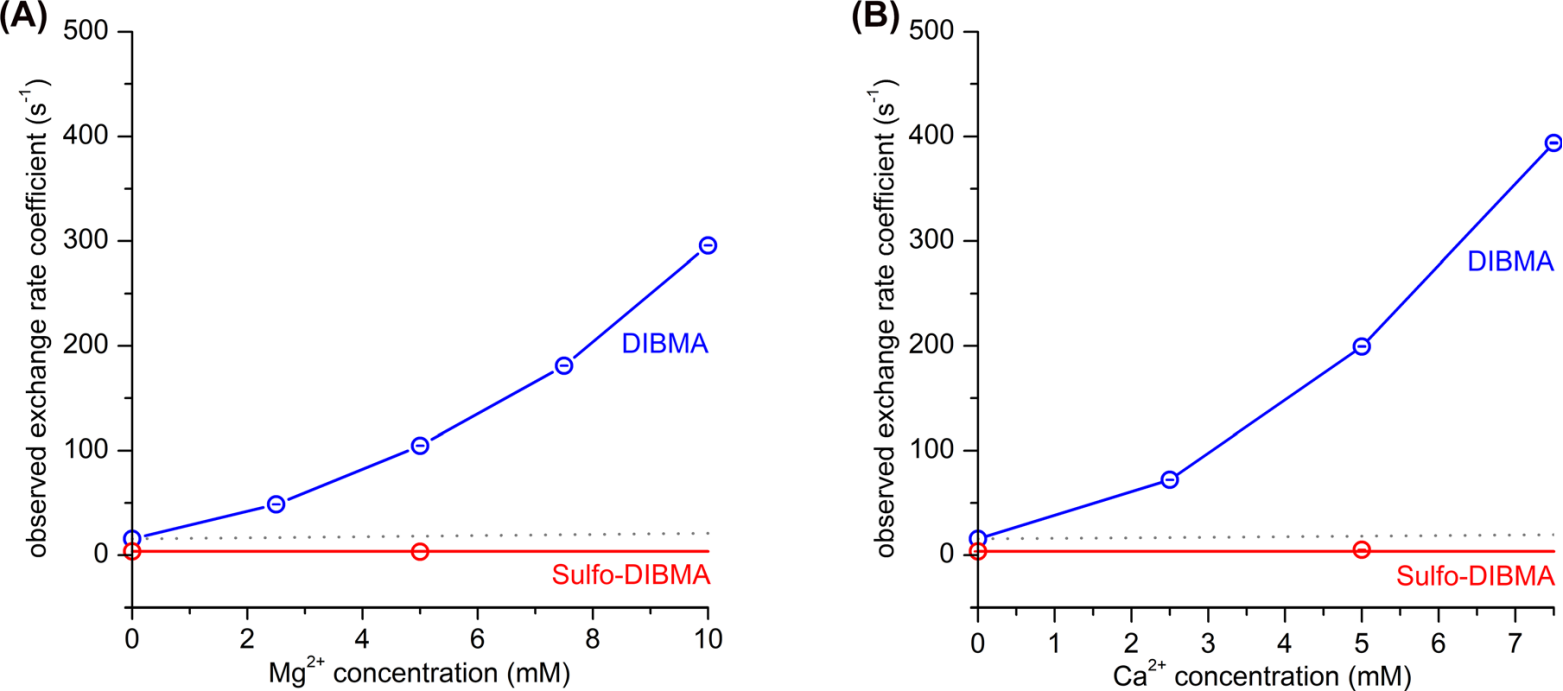
Divalent cations do not affect lipid exchange among Sulfo-DIBMA nanodiscs. Observed rate coefficients (*k*_obs_) characterizing overall lipid exchange among Sulfo-DIBMA nanodiscs (this work; measured at a final concentration of unlabeled lipid of 2.5 mM) and among DIBMA nanodiscs (Danielczak et al. 2019) as functions of (A) Mg^2+^ concentration and (B) Ca^2+^ concentration. Also shown are simulations of *k*_obs_ as function of divalent cation concentration considering exclusively Coulombic screening for DIBMA nanodiscs according to Equation (5) (dashed grey lines) (Danielczak et al. 2019). Error bars (within circles) indicate 95% confidence intervals.

Our results show that lipid exchange among Sulfo-DIBMA nanodiscs is not noticeably affected by the presence of either Mg^2+^ (Figure 5A) or Ca^2+^ (Figure 5B). These results contrast strongly with those for DIBMA nanodiscs, for which *k*_obs_ was shown to increase at least 10-fold when the concentration of either Mg^2+^ or Ca^2+^ was increased from 0 to 5 mM (Danielczak et al. 2019). Our findings further demonstrate that the electroneutrality of Sulfo-DIBMA stabilizes it against changes in ion concentration, not only for monovalent but also divalent cations, contrasting with the behavior of polyanionic polymers such as DIBMA and SMA.

## 3 Discussion

In this study, we aimed to determine how the electroneutrality of Sulfo-DIBMA nanodiscs influences their lipid-exchange kinetics and mechanisms. In addition, we posed the hypothesis that lipid exchange among the electroneutral Sulfo-DIBMA nanodiscs should be unaffected by changes in ion concentration.

### 3.1 Relatively slow lipid exchange among Sulfo-DIBMA nanodiscs may be due to a high free-energy barrier to dehydration

Our finding that Sulfo-DIBMA nanodiscs exchange lipids on a similar timescale as DIBMA nanodiscs—but much more slowly than Glyco-DIBMA nanodiscs—cannot be simply explained by differences in charge density alone. Considering only such simple coulombic arguments (Danielczak and Keller 2018; Grethen et al. 2018), one would expect collisional lipid exchange to be much faster for electroneutral Sulfo-DIBMA nanodiscs than for highly negatively charged DIBMA nanodiscs. Therefore, there must be another mechanism at play here. We propose that the main reason for the slower lipid exchange among Sulfo-DIBMA nanodiscs is the dehydration of the sulfobetaine groups.

Dehydration is necessary for the transient fusion of two or three nanodiscs upon collision. Since the dehydration of sulfobetaine groups is thermodynamically unfavorable (Racovita et al. 2021), it poses a high free-energy barrier that slows down collisional lipid exchange. Our data suggest that this free-energy barrier encountered by electroneutral Sulfo-DIBMA nanodiscs is about as high as that encountered by polyanionic DIBMA nanodiscs. This interpretation is supported by the similar *k*_bi_ and *k*_ter_ values determined for Sulfo-DIBMA and DIBMA nanodiscs. Glyco-DIBMA, by contrast, has fewer carboxylate groups than DIBMA and, instead, carries sugar pendant groups. Some of these sugar groups will also need to be dehydrated for transient fusion to take place, but dehydrating a sugar group is thermodynamically less costly than dehydrating a sulfobetaine group (Racovita et al. 2021). Such a difference in dehydration penalty might explain why collisional lipid exchange among Sulfo-DIBMA nanodiscs is slower than among Glyco-DIBMA nanodiscs.

### 3.2 Lipid exchange among Sulfo-DIBMA nanodiscs is unaffected by changes in ion concentration

Since Sulfo-DIBMA nanodiscs are electroneutral, we hypothesized that coulombic repulsion and counterion association should not affect the lipid-exchange kinetics. Indeed, our results confirmed that lipid exchange among Sulfo-DIBMA nanodiscs is not affected by the presence of mono- or even divalent cations. This differs dramatically from the results obtained for highly anionic DIBMA (Danielczak et al. 2019).

Our results show that lipid exchange among Sulfo-DIBMA is relatively slow and that this exchange is largely unaffected affected by the presence of cations at physiologically relevant concentrations. This robustness towards the presence of ions is in line with previous findings for zwitterionic nanodisc-forming polymers (Janson et al. 2021). This lipid-exchange behavior renders Sulfo-DIBMA nanodiscs a promising membrane mimetic for studying charge-sensitive interactions, especially since Sulfo-DIBMA nanodiscs are colloidally highly stable at high NaCl, Mg^2+^, and Ca^2+^ concentrations (Glueck et al. 2022). Two examples of such charge-sensitive interactions that have already been examined using Sulfo-DIBMA nanodiscs are firstly, the differential binding reactions of *α*-synuclein and the peptidic adrenocorticotropic hormone (ACTH) to zwitterionic versus anionic phospholipid bilayers (Glueck et al. 2022), and secondly, cell-free translation (Glueck et al. 2022). Contrastingly, the presence of divalent cations has been reported to have detrimental effects on the colloidal stability of DIBMA nanodiscs. For example, in the presence of Mg^2+^ and Ca^2+^ at physiologically relevant concentrations, the formation of DIBMA nanodiscs is less efficient, the nanodiscs are colloidally unstable, and their lipid exchange is noticeably faster (Danielczak et al. 2019; Danielczak and Keller 2018). Therefore, Sulfo-DIBMA nanodiscs are a more controllable membrane mimetic, which does not interfere with charge-sensitive interactions and analyses.

Taken together, our results show that Sulfo-DIBMA nanodiscs exchange lipids more slowly than Glyco-DIBMA nanodiscs but at a similar rate as DIBMA nanodiscs. Unlike DIBMA nanodiscs, however, lipid exchange among Sulfo-DIBMA nanodiscs is independent of ionic strength and counterion concentration. This robust lipid exchange together with their stability in the presence of ions and their compatibility with charge-sensitive interactions and analyses will allow Sulfo-DIBMA nanodiscs to be used in diverse settings for membrane-protein research.

## 4 Materials and methods

### 4.1 Materials

Sulfo-DIBMA was obtained from Glycon Biochemicals (Luckenwalde, Germany). DMPC was a kind gift from Lipoid (Ludwigshafen, Germany). NBD-PE and Rh-PE were purchased from Avanti Polar Lipids (Alabaster, USA). Tris(hydroxymethyl)aminomethane (Tris), NaOH, NaCl, HCl, MgCl_2_, CaCl_2_, and CHCl_3_ were purchased from Carl Roth (Karlsruhe, Germany) and Sigma–Aldrich (Steinheim, Germany). All chemicals were purchased in the highest purity available.

### 4.2 Dynamic light scattering (DLS)

DLS measurements were performed on a Zetasizer Nano S90 (Malvern Panalytical, Malvern, UK) equipped with a He–Ne Laser emitting at 633 nm. 60-µL samples were thermostatted at 35 °C and measured in 3 mm × 3 mm quartz cuvettes (Hellma Analytics, Müllheim, Germany). DLS measurements were performed as described previously (Glueck et al. 2022). Effects of buffer components on viscosity and refractive index of the solvent were accounted for during data analysis (Glueck et al. 2022). Fluorescently labeled samples were discarded after DLS measurements because bleaching could disturb later TR-FRET measurements. Unlabeled samples were reused for TR-FRET measurements.

### 4.3 TR-FRET

TR-FRET was measured on an SF.3 stopped-flow apparatus (Applied Photophysics, Leatherhead, UK) equipped with a (470 ± 10) nm light-emitting diode with a power output of 20 mA. The excitation beam was attenuated by a TechSpec OD2 band-pass filter (Edmund Optics, Karlsruhe, Germany) to avoid photobleaching of fluorescently labeled lipids. TR-FRET was monitored at (528 ± 15) nm using a photomultiplier at a 90° angle with a TechSpec OD6 band-pass filter (Edmund Optics, Karlsruhe, Germany). The drive syringes, tubes, and quartz-glass cell were thermostatted at 35 °C.

### 4.4 Experimental protocols

All steps involving the exposure of labeled lipids were performed under low-light conditions to avoid photobleaching. For a detailed and general protocol, see Danielczak and Keller (2020).

*Buffers:* Buffers 0 to 4 were prepared using triple-distilled water (dddH_2_O) according to Table 2, adjusted to pH 7.4 using 1 M NaOH or 1 M HCl, and sterile-filtered.

**Table 2:** Salt and buffer concentrations of buffers 0–4.

Buffer	[Tris] (mM)	[NaCl] (mM)	[MgCl_2_] (mM)	[CaCl_2_] (mM)
0	50	200	–	–
1	50	50	–	–
2	50	500	–	–
3	50	200	5	–
4	50	200	–	5

*Unlabeled Sulfo-DIBMA/DMPC nanodiscs:* To produce unlabeled nanodiscs, a DMPC stock suspension (10 mM) and a Sulfo-DIBMA stock solution (∼25 mg/mL) were prepared in buffer and dispersed in an ultrasonic bath until the suspension appeared homogeneous. The Sulfo-DIBMA stock was sterile-filtered and, if necessary, adjusted to pH 7.0. The concentration of Sulfo-DIBMA was determined via refractometry according to the following equation:

(1) $$c_{sulfo-DIBMA}=\frac{{RI}_{stock}-{RI}_{buffer}}{dn/d\rho }$$

Here, RI_stock_ and RI_buffer_ are the refractive indices of the Sulfo-DIBMA stock solution and buffer 0, respectively, and d*n*/d*ρ* = 0.1174 L kg^−1^ is the refractive index increment of Sulfo-DIBMA. The DMPC stock suspension was subjected to six freeze–thaw cycles and then mixed with Sulfo-DIBMA and one of buffers 1–4 to obtain 1.5 mL of unlabeled nanodiscs (*c*_L_ = 5 mM) at a polymer/lipid mass ratio of 2.00. For buffer 0, 1.3 mL of unlabeled nanodiscs (*c*_L_ = 1.25 mM, 2.5 mM, 5 mM, 10 mM, 20 mM, or 30 mM) was produced by diluting unlabeled nanodisc stock solution (*c*_L_ = 30 mM) having a polymer/lipid mass ratio of 2.00. After solubilizing the lipids at 35 °C and 800 rpm on an Eppendorf ThermoMixer F2.0 for at least 16 h, DLS measurements were performed to confirm the formation of nanodiscs. The average hydrodynamic size of unlabeled nanodiscs as determined by DLS and the associated standard deviation amounted to (8.65 ± 0.25) nm (*n* = 5). This size agrees well with values previously determined by DLS (Glueck et al. 2022), microfluidic diffusional sizing (MDS) (Glueck et al. 2022), negative-stain electron microscopy (nsEM) (Glueck et al. 2022; Janson et al. 2022), and cryoEM (Janson et al. 2022).

*Labeled Sulfo-DIBMA/DMPC nanodiscs:* For labeled nanodiscs in buffer 0, stock solutions of NBD-PE (0.5 mM) and Rh-PE (0.5 mM) in CHCl_3_ and a stock solution of DMPC (15 mM) in CHCl_3_ were prepared. These were mixed to obtain a suspension containing DMPC (9.6 mM), Rh-PE (0.2 mM), and NBD-PE (0.2 mM) in buffer 0. CHCl_3_ was removed from the mixed lipid solution by passing a nitrogen stream over the solution and then storing the sample in a vacuum desiccator for at least 16 h. The resulting dry lipid film was resuspended in the respective buffer, and six freeze–thaw cycles were performed. A suspension of labeled nanodiscs (10 mL, concentration of labeled lipids *c*_L_° = 0.5 mM) was prepared by mixing the resuspended lipids with the Sulfo-DIBMA stock solution and buffer 0. For labeled nanodiscs in buffers 1–4, a labeled nanodisc suspension (1.5 mL; *c*_L_° = 0.5 mM) was prepared analogously. Solubilization was carried out as described above (35 °C, 800 rpm on an Eppendorf ThermoMixer F2.0, 16 h) using a polymer/lipid mass ratio of 2.00. DLS measurements were carried out to confirm the formation of nanodiscs. The average hydrodynamic size of labeled nanodiscs as determined by DLS and the associated standard deviation amounted to (8.90 ± 1.47) nm (*n* = 5), again in good agreement with previous values determined for Sulfo-DIBMA nanodiscs by DLS and other methods (Glueck et al. 2022; Janson et al. 2022).

*Stopped-flow measurements*: For the TR-FRET measurements, three initial washing steps were performed with 20% ethanol in dddH_2_O, dddH_2_O, and the buffer (0–4) used in the following experiment. Each washing step consisted of 12 injections and the recording of one data point every 0.1 s. For measurements in buffer 0, no in-between washing steps were needed since measurements were performed from lowest to highest lipid concentration (unlabeled nanodiscs). Three washing steps with dddH_2_O and then with the new buffer were performed whenever the buffer was changed.

Samples containing unlabeled and labeled nanodiscs were loaded using 2-mL syringes. Loading approximately 1 mL of the samples allowed for several individual injections to be made from each sample. For equilibration, three injections were made, recording one data point every 0.1 s. To estimate the total measurement time needed, an initial measurement was performed. This initial measurement consisted of a single injection followed by recording 10,000 data points over 1000 s. The actual measurement then consisted of five injections, each of which was followed by recording 10,000 datapoints over 5400 s, 3600 s, 3000 s, 2700 s, 1800 s, 900 s at final unlabeled lipid concentrations of 0.625 mM, 1.25 mM, 2.5 mM, 5 mM, 10 mM, and 15 mM, respectively.

*Data analysis:* Data analysis was performed in MS Excel 2016 (Microsoft Corporation, Redmond, USA), using the Solver add-in for nonlinear least-squares fitting, as described previously (Danielczak and Keller 2020; Kemmer and Keller 2010). Data were plotted in Origin 8.1. (OriginLab, Northampton, USA).

### 4.5 Theoretical background

*Kinetics of lipid exchange among nanodiscs:* The overall lipid exchange as quantified by *k*_obs_ can be accounted for by two major mechanisms (Cuevas Arenas et al. 2017; Danielczak and Keller 2020). The first mechanism—diffusional exchange—includes desorption and inter-particle diffusion of lipid monomers through the aqueous phase surrounding the nanodisc (Nichols 1985; Nichols and Pagano 1981, 1982). This slow process is described by a saturable kinetic model with the rate coefficient *k*_diff_ and dominates at low lipid concentrations (Cuevas Arenas et al. 2016; Fullington et al. 1990; Fullington and Nichols 1993; Nichols 1985; Nichols and Pagano 1981, 1982). The second mechanism—collisional exchange—is based on nanoparticle collisions, resulting in a second- or third-order reaction reflecting binary or ternary collisions, respectively (Fullington et al. 1990; Fullington and Nichols 1993; Jones and Thompson 1990; Nichols 1988). Since binary collisions, represented by the rate coefficient *k*_bi_, follow second-order kinetics, they dominate at intermediate lipid concentrations, whereas ternary collisions, represented by the rate coefficient *k*_ter_, are increasingly significant as lipid concentration rises (Fullington et al. 1990; Fullington and Nichols 1993; Jones and Thompson 1990; Nichols 1988).

The overall observed rate coefficient of lipid exchange (*k*_obs_) can be accounted for by the rate coefficients *k*_diff_, *k*_bi_, and *k*_ter_, together with the final concentrations of lipid in unlabeled nanodiscs (0.625 mM ≤ *c*_L_ ≤ 15 mM) and of lipid in labeled nanodiscs (*c*_L_°; here, *c*_L_° = 0.25 mM) (Danielczak and Keller 2020). Consequently, determining the concentration dependence of *k*_obs_ allows the mechanism-specific rate coefficients *k*_diff_, *k*_bi_, and *k*_ter_ to be deduced, yielding the contribution of each mechanism to the overall lipid exchange (Cuevas Arenas et al. 2017; Danielczak and Keller 2020).

(2) $$k_{obs}\left(c_{L}\right)=\frac{k_{diff}c_{L}}{c_{L}^{{}^{\circ}}+c_{L}}+k_{bi}c_{L}+k_{ter}c_{L}^{2}$$

*Investigating lipid-exchange kinetics among nanodiscs with TR-FRET spectroscopy*: TR-FRET spectroscopy is a highly sensitive, fast, and efficient technique for studying lipid-exchange kinetics among nanoparticles, including polymer-encapsulated nanodiscs (Cuevas Arenas et al. 2017; Danielczak et al. 2019; Danielczak and Keller 2018, 2020; Grethen et al. 2018). In TR-FRET lipid-exchange experiments, two fluorescently labeled lipids—acting as a FRET donor/acceptor pair—form a sensitive reporter system for detecting changes in lipid composition over time (Danielczak and Keller 2020). In this case, NBD-PE as donor and Rh-PE as acceptor form a suitable FRET pair for this purpose, with a Förster distance of *R*_0_ = 6.6 nm (Danielczak and Keller 2020; Wolf et al. 1992). Fast mixing of nanodiscs containing both FRET donor and acceptor lipids with unlabeled nanodiscs in a stopped-flow apparatus dilutes the labeled lipids with the unlabeled lipid background (Danielczak and Keller 2020). Thus, mixing between labeled and unlabeled nanodiscs increases the average distance between FRET donor and acceptor lipids, reducing the FRET efficiency, as reflected in de-quenching of the donor (Danielczak and Keller 2020). The resulting increase in fluorescence intensity as a function of time *t* after mixing (*F* (*t*)), can be described using the observed lipid-exchange rate coefficient (*k*_obs_), the fluorescence intensity at *t*_0_ (*F*_0_), and the fluorescence intensity at *t*_∞_ (*F*_∞_) (Danielczak and Keller 2020).

(3) $$F\left(t\right)=F_{\infty }+e^{-k_{obs}t}\;\left(F_{0}-F_{\infty }\right)$$

Using nonlinear least-squares fitting of the TR-FRET fluorescence data, the lipid-exchange-rate coefficients can then be determined (Danielczak and Keller 2020; Kemmer and Keller 2010). The theoretical background is described in detail in previous publications (Cuevas Arenas et al. 2017; Danielczak et al. 2019; Danielczak and Keller 2018, 2020; Grethen et al. 2018). 95% confidence intervals were calculated by sensitivity analysis (Danielczak and Keller 2020; Kemmer and Keller 2010). To this end, one of the fitting parameters was fixed at a non-optimal value before the remaining fitting parameters were subjected to non-linear least-squares optimization. The monotonic increase in the sum of squared residuals (SSR) with increasing deviation from the best-fit value was then used in conjunction with Fisher’s *F* distribution to estimate the 95% confidence intervals, as detailed elsewhere (Kemmer and Keller 2010).

*Impact of coulombic repulsion on lipid exchange among nanodiscs*: Electrostatic effects can noticeably affect collisional lipid exchange (Danielczak and Keller 2018, 2020; Grethen et al. 2018). Specifically, coulombic repulsion between nanodiscs can be reduced by increasing the ionic strength of the surrounding aqueous phase, since the cations in solution will somewhat “screen” the negative charges on the polymer (Danielczak and Keller 2018, 2020; Grethen et al. 2018). This screening effect can be quantified by combining the so-called extended Debye–Hückel limiting law with the kinetic salt effect (Danielczak and Keller 2018, 2020; Grethen et al. 2018):

(4) $$\log\left(\frac{k_{bi}}{M^{-1}s^{-1}}\right)=\;\log\left(\frac{k_{bi}^{0}}{M^{-1}s^{-1}}\right)-2\left(\frac{-Az^{2}\sqrt{I}}{1+Br\sqrt{I}}\right)$$

Parameters that can be determined by nonlinear least-squares fitting of this extended Debye–Hückel limiting law include the lipid-exchange rate coefficient for lipid exchange via binary collisions at vanishing ionic strength (*k*_bi_^0^; at *γ* = 1) and the effective nanodisc charge (*z*) (Danielczak and Keller 2020). *A* and *B* are constants, which are defined as (0.516 L^1/2^ mol^−1/2^) and (3.30 nm^−1^ L^1/2^ mol^−1/2^) respectively (Danielczak and Keller 2020). Parameters derived from the experimental setup, are the ionic activity coefficient (*γ*), the lipid-exchange rate coefficient for lipid exchange via binary collisions (*k*_bi_), the *I* (ionic strength), and the nanodisc radius in (nm) (*r*). 95% confidence intervals were again obtained by sensitivity analysis using Fisher’s *F* distribution (Kemmer and Keller 2010).

*Impact of counterion association on lipid exchange among nanodiscs*: Coulombic repulsion can also be attenuated by specific counterion association, which is particularly pronounced for multivalent ions, such as Mg^2+^ and Ca^2+^ (Bergethon 1998; Danielczak and Keller 2020; Emyr Alun Moelwyn-Hughes 1963). The equation below allows the effects of coulombic repulsion and specific counterion association to be distinguished from one another (Danielczak and Keller 2020). The effective nanodisc charge number (*z*) can then be determined via nonlinear least-squares fitting of the following equation:

(5) $$\log\left(\frac{k_{bi}^{M^{2+}}}{k_{bi}^{w/o}}\right)=2Az^{2}\left(\frac{\sqrt{I_{M^{2+}}}}{1+Br\sqrt{I_{M^{2+}}}}-\frac{\sqrt{I_{0}}}{1+Br\sqrt{I_{0}}}\right)$$

Parameters derived from the experimental setup, are the lipid-exchange rate coefficient for lipid exchange via binary collisions in the presence of divalent metal ions ( $k_{bi}^{M^{2+}}$ ), the lipid-exchange rate coefficient for lipid exchange via binary collisions without divalent metal ions (*k*_bi_^w/o^), the ionic strength in presence of divalent metal ions (*I*_M2+_), the ionic strength without divalent metal ions (*I*_0_), and the nanodisc radius in (nm) (*r*) (Danielczak and Keller 2020). Again, *A* and *B* are constants, which are defined as (0.516 L^1/2^ mol^−1/2^) and (3.30 nm^−1^ L^1/2^ mol^−1/2^) respectively (Danielczak and Keller 2020). 95% confidence intervals were again obtained by sensitivity analysis using Fisher’s *F* distribution (Danielczak and Keller 2020; Kemmer and Keller 2010).
